# Synergistic Anti-Cancer Effects of Isocnicin and Radiotherapy in Glioblastoma: A Natural Compound’s Potential

**DOI:** 10.3390/biomedicines12122793

**Published:** 2024-12-09

**Authors:** Effrosyni Tsafa, Kyriakos Dimitriadis, Lamprini Kalampoki, Panagiota Papapetrou, Pavlos A. Georgalis, Georgios Bozios, Chrissa Sioka, Pericles Tsekeris, Athanassios P. Kyritsis, George A. Alexiou, Diamanto Lazari

**Affiliations:** 1 Neurosurgical Institute, University of Ioannina, 451 10 Ioannina, Greece; efitsafa86@yahoo.gr (E.T.); lamprini.kalampoki@gmail.com (L.K.); papapetrou.p6@gmail.com (P.P.); pavlosgeorgalis@gmail.com (P.A.G.); csioka@yahoo.com (C.S.); thkyrits@uoi.gr (A.P.K.); 2 Laboratory of Pharmacognosy, School of Pharmacy, Faculty of Health Sciences, Aristotle University of Thessaloniki, 541 24 Thessaloniki, Greece; kmdimitri@pharm.auth.gr (K.D.); dlazari@pharm.auth.gr (D.L.); 3 Department of Radiation Oncology, University of Ioannina, 451 10 Ioannina, Greece; gbozios@uhi.gr (G.B.); ptsekeri@uoi.gr (P.T.); 4 Department of Neurosurgery, University of Ioannina, 451 10 Ioannina, Greece

**Keywords:** cancer, glioblastoma, temozolomide, cytostatic agents, cell cycle, flow cytometry, prognosis, antineoplastic agents, isocnicin, radiation

## Abstract

Background/Objectives: Glioblastoma (GBM) is the most aggressive type of brain tumor in adults. Currently, the only treatments available are surgery, radiotherapy, and chemotherapy based on temozolomide (TMZ); however, the prognosis is dismal. Several natural substances are under investigation for cancer treatment. 8α-O-(3,4-dihydroxy-2-methylenebutanoyloxy) dehydromelitensine (Isocnicin) is a natural compound derived from *Centaurea* species and was found to exhibit cytostatic/cytotoxic effect against different cell lines. In this study, we investigated the anti-glioma effects of isocnicin in U87 and T98 glioblastoma cell lines, as well as the effects of combined treatment with radiotherapy. Methods: Cell viability was evaluated with the trypan blue exclusion assay, cell cycle distribution was examined using flow cytometry, and the effects of the combination treatment were analyzed with CompuSyn software(1.0). Results: The result showed that isocnicin significantly reduced cell viability in U87 and T98 cell lines in a dose-dependent manner and IC_50_ values were calculated. Administration of isocnicin alone induced both S and G2/M cell cycle arrest in U87 and T98 cells in a dose-dependent manner. Moreover, when cells were treated with increasing concentrations of isocnicin, followed by 2 or 4 Gy of radiation, the percentage distribution of the cells in the G2/M phase was increased considerably in both U87 and T98 cell lines. Conclusions: Here, we show for the first time that co-treatment of isocnicin with radiation exerts a synergistic antiproliferative effect in glioblastoma cell lines. Natural compounds are promising for glioblastoma treatment. Further studies will be necessary to unravel isocnicin’s mechanism of action and its synergistic effect with radiation on glioblastoma treatment.

## 1. Introduction

**Figure 1:**
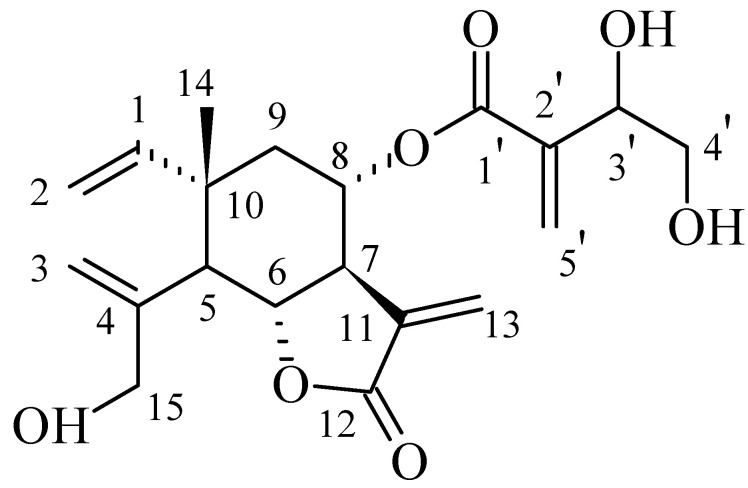
Structure of 8α-O-(3′,4′-dihydroxy-2′-methylenebutanoyloxy)-dehydromelitensine (Isocnicin).

Glioblastoma (GBM) is the most aggressive type of primary brain tumor in adults and has a very poor prognosis. Currently, the only treatments available are surgery, radiotherapy, and chemotherapy based on temozolomide (TMZ) [1]. However, the mean survival period for patients is only 14-16 months, and only a few GBM patients survive for 5 years or more. Moreover, despite treatment, the neoplasm almost always recurs within a few months [2,3]. The presence of the blood–brain barrier (BBB), the heterogeneity of the tumor and the infiltration of glioblastoma cells into the surrounding tissues has, so far, prevented the development of an effective treatment [4,5]. Since ancient times, natural compounds derived from medicinal plants have been utilized. In addition to being used to treat a variety of diseases, natural products are increasingly being used in drug development and discovery. According to epidemiological research, consuming fruits and vegetables decreases the risk of developing cancer. Recently, research has focused on the investigation of natural substances, such as soy, curcumin, resveratrol, and retinoids to treat GBM [6,7]. A clinical trial on tolerability, safety, and efficacy of liposomal curcumin in combination with radiotherapy and Temozolomide in patients with newly diagnosed high-grade gliomas is currently recruiting patients (NCT05768919). Thus, there is a great interest in natural compounds. Another natural product known for its cytotoxic and cytostatic properties is 8α-O-(3,4-dihydroxy-2-methylenebutanoyloxy)-dehydromelitensine (Isocnicin). *Centaurea* sp., a member of the Asteraceae family found in West Asia and the Mediterranean region, contains the natural substance isocnicin. Previously, five human cell lines (DLD1, SF268, MCF7, H460, and OVCAR3) were used to test the in vitro cytotoxic/cytostatic activity of sesquiterpene lactones that were extracted from the aerial portions of different species of the genus *Centaurea*. Among the various compounds, isocnicin was the most active, exhibiting considerable growth-inhibiting activity against three of the cell lines tested [8]. Its chemical structure is shown in Figure 1 and its molecular weight was found to be 378. Since the molecular weight of isocnicin is 378 Daltons it might be able to cross the blood–brain barrier (BBB) [9].

Given that radiotherapy is a standard treatment for GBM patients, isocnicin’s possible radiosensitizing effect in human glioma cell lines deserves investigation. The dose range and timing of isocnicin administration are both critical when combined with irradiation. In the current study, we investigated the isocnicin’s anti-cancer effects in glioblastoma cell lines in combination with radiotherapy.

## 2. Materials and Methods

In order to evaluate the effect of isocnicin on glioblastoma cells we utilized the trypan blue exclusion test for the viability assays, flow cytometry for cell cycle analysis and radiation delivery was performed, using X-rays generated by a linac 6 MV accelerator (Varian Medical Systems) as described previously.

### 2.1. Isolation and Identification of 8α-O-(3′,4′-dihydroxy-2′-methylenebutanoyloxy)-dehydromelitensine (Isocnicin)

Dr. Shahid Farooq, Plant Taxonomist of the Pakistan Council of Scientific and Industrial Research-PCSIR, Peshawar, verified that 8α-O-(3′,4′-dihydroxy-2′-methylenebutanoyloxy)-dehydromelitensine (isocnicin) was a pure oily compound that was extracted from the ethyl acetate extract of *Centaurea bruguieriana* subsp. *belangeriana* (DC.) Bornm., which was collected from the suburbs of Peshawar, Pakistan. Under the identifier CA-001-04, a voucher specimen was placed in the Herbarium at the PCSIR Laboratories in Peshawar. A mixture of cyclohexane:diethyl ether:methanol 1:1:1 was used to finely grind and extract the 1.5 kg of air-dried aerial parts of *C. bruguieriana* subsp. *belangeriana* (DC.) Bornm. at room temperature, yielding 80.46 g of extract. After being redissolved using the same combination, the extract was rinsed with brine. Ethyl acetate (EtOAc) was used to extract the aqueous fraction (Organic phase B, 25.81 g). To separate isocnicin from the extract, chromatographic techniques such vacuum layer chromatography (VLC), thin layer chromatography (TLC), and column chromatography (CC) were chosen.

With gradient elution using solvent combinations, organic phase B was fractionated using vacuum liquid chromatography (VLC) (10.0 × 7.0 cm) on silica gel (Merck 60H, Art. 7736). Eleven fractions, each containing 300 mL, were obtained (A-L). A total of 14 fractions (GA-GO) were obtained by eluting fraction G (1.47 g) with ethyl acetate:acetone (75:25) and then subjecting it to CC (18.0 × 3.0 cm) on silica gel (Merck 60H, Art. 9385) using dichloromethane (CH_2_Cl_2_)–methanol (MeOH) solutions of increasing polarity as eluents. It was determined that the isocnicin was fraction GH (42.0 mg) that eluted with CH_2_Cl_2_-MeOH (95:5). The fractions’ quality was managed using TLC. The TLC was performed using a cellulose (Merck, Art. 5552) stationary phase on aluminum foil (20 × 20 cm, 0.1 mm) and a silica gel (Kie-selgel F254, Merck, Art. 5554, Merck GLOBAL, Athens, Greece) with a fluorescent marker. The TLC plates were developed using solvent combinations that were suitable for each fraction group. Lastly, vanillin-H_2_SO_4_ (1:1) was sprayed on the silica gel TLC plates [10], and Naturstoffreagenz A was sprayed on the cellulose plates [11]. Using 1D NMR (Nuclear Magnetic Resonance) research, isocnicin was identified and verified (^1^H). The AG-ILENT DD2 500 (500.1 MHz for ^1^H-NMR) spectrometer was used to record the ^1^H-NMR in CD_3_OD and CDCl_3_. Relative to TMS (tetramethylsilane), chemical shifts are expressed in δ (ppm) values (3.31 ppm for CD_3_OD and 7.24 ppm for CDCl_3_ for ^1^H-NMR). Samples from our collection and/or data published in the literature were compared with the data of isolated and identified isocnicin (Tables S1 and S2 and Figures S1 and S2) [12].

### 2.2. Cell Lines and Conditions for Treatment

The human glioma cell lines U87 and T98 (Manassas, VA, USA) were supplied by Dr. W. K. Alfred Yung (Department of Neuro-Oncology, M.D. Anderson Cancer Center, Houston, Texas, USA) and ATCC, respectively. All of the cell lines were cultivated in Dulbecco’s modified Eagle’s medium (DMEM, Gibco BRL, Life Technologies, Grand Island, NY, USA), supplemented with 1% penicillin-streptomycin and 10% fetal bovine serum (FBS) (Gibco BRL). Every cell line was incubated in a humidified atmosphere with 5% CO_2_ at 37 °C. Dimethyl sulfoxide (DMSO) was used to dilute isocnicin from stock solution (1 mM) to the final concentration using culture medium prior to each experiment. Isocnicin was administered to malignant glioma cell cultures both with and without radiation.

### 2.3. The Viability Test

About 10,000 cells were plated in 24-well plates, and after 24 h, they were subjected to escalating isocnicin doses (10–120 μM) in order to conduct a Trypan Blue exclusion experiment. Using the Trypan blue exclusion test, cell viability was assessed after 72 h [13,14]. The data presented represent the mean of the experiment, which was conducted at least three times.

### 2.4. Flow Cytometric Analysis of DNA Cell Cycle

About 20,000 cells were seeded in 12-well plates for the DNA cell cycle study, and after 24 h, the cells were exposed to isocnicin at 0.5× IC50, IC50, and twice IC50 concentrations for another 72 h. Untreated cells were used as a negative control. All samples were tested 3 times in at least 3 independent experiments. Cells were then extracted after trypsin treatment and maintained at 37 °C for 20 min with a PI working solution (50 g/mL PI, 20 mg/mL RNase A, and 0.1% Triton X-100). Next, cells were rinsed with PBS solution. The PI fluorescence of 10,000 individual nuclei was calculated. The cell cycle fractions of the cells in G0/G1, S, G2/M, and sub-G0/G1 phase were analysed. Data on PI fluorescence were gathered using a flow cytometer (Omnicyt, Cytognos S.L., Grand Island, NY, USA), and were examined using the software programs MedCalc and GraphPad Prism version 6 (Trial version) [15].

### 2.5. Radiation and Isocnicin Combined Therapy

Isocnicin was administered to malignant glioma cell cultures either with or without radiation. U87 and T98 cells were grown on 12-well plates, treated with isocnicin after 24 h, in concentrations ranging from 0, 3.5, 7, 14, 21, 28 μΜ for U87 cells to 0, 13.5, 27, 54, 81, 108 μΜ for T98 cells, respectively, and then exposed to doses of 2 or 4 Gy of radiation 2 h later, as previously described [16]. Trypan blue exclusion test was used to assess viability and DNA cell cycle analysis using Flow Cytometry was performed.

### 2.6. Statistical Analysis

Standard deviation (SD) ± mean was used to express the data. Regression analysis using GraphPad Prism software (v. 8.0.0, San Diego, CA, USA, Trial Version) was used to determine IC50 values. The post hoc Tukey test and one-way ANOVA were used to evaluate group comparisons. Significant differences were defined as *p* < 0.05.

## 3. Results

### 3.1. Calculation of Viability and IC50 of GBM Cells After Isocnicin Treatment

**Figure 2:**
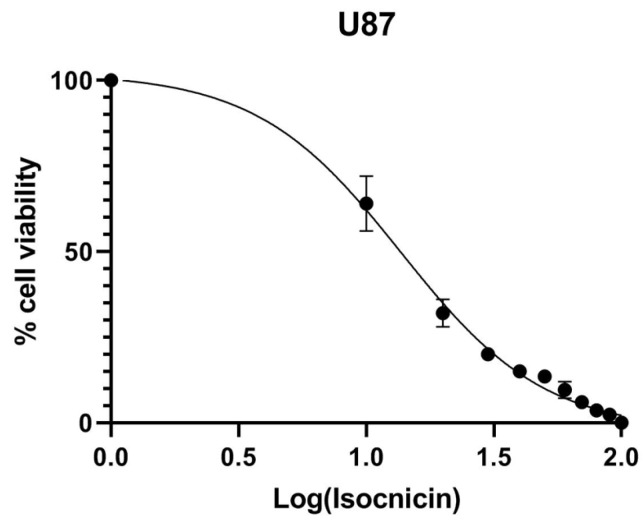

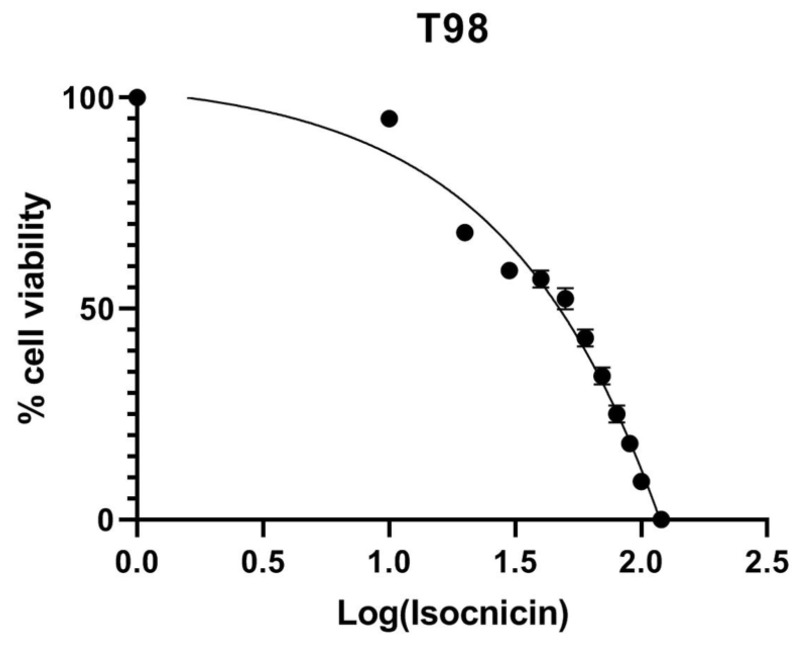
Cytotoxic effect of isocnicin on U87 and T98 cell lines, 72 h after treatment. Cells were quantified by staining with Trypan Blue and data shown are the means (±S.D.) from 3 different experiments. IC50 values were determined using the GraphPad Prism version 8 non-linear regression analysis model.

**Figure 3:**
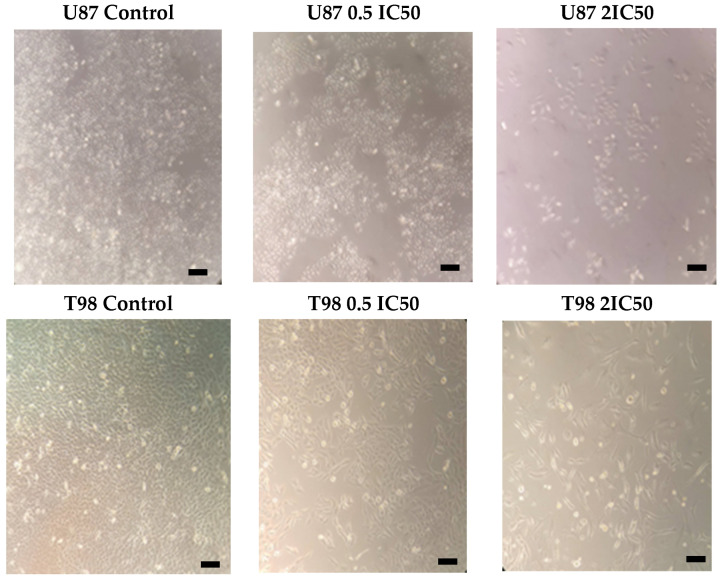
Microscopy (10×) observation of the U87 and T98 cell lines after treatment with isocnicin for 72 h (Scale bars = 50 μΜ).

T98 and U87 cells were cultured with increasing concentrations of isocnicin for 72 h to test the susceptibility of GBM cells to isocnicin. In U87 and T98 cells, the IC50 of isocnicin for viability reduction was 14 μM and 54 μM, respectively (Figure 2). With increasing isocnicin concentrations, cells underwent changes such as cell shrinkage and cell death, visible under a microscope (Figure 3).

### 3.2. Combined Effects of Isocnicin and Radiation on Glioblastoma Cells

Isocnicin was used at concentrations ranging from 3.5–28 μM to 13.5–108 μM in U87 and T98 cells, respectively, and radiation was administered at doses of 2 or 4 Gy. CompuSyn software (1.0) was used to determine the synergism or antagonism between isocnicin and radiation. CompuSyn calculates the Combination index (CI: a quantitative measure of the degree of drug interaction in terms of synergism and antagonism for a given endpoint of the effect measurement) for each drug combination, where CI < 1 indicates synergism (greater than expected additive result), CI = 1 is the additive result and CI > 1 indicates antagonism (smaller than expected additive result). The effects of isocnicin in combination with radiation are summarized in Table 1 and Table 2. In U87 cells, isocnicin and radiation were synergistic for most of the combinations tested, with the highest synergy observed when isocnicin was administered at 21 μΜ combined with 4 Gy radiation (CI: 0.81). The highest synergy was observed at higher isocnicin concentrations in T98 cells, especially at 108 μM, probably because these cells are resistant to both chemotherapy regimens and radiotherapy. Overall, isocnicin and irradiation showed in several cases a strong synergy relationship in both cell lines.

**Figure 4:**
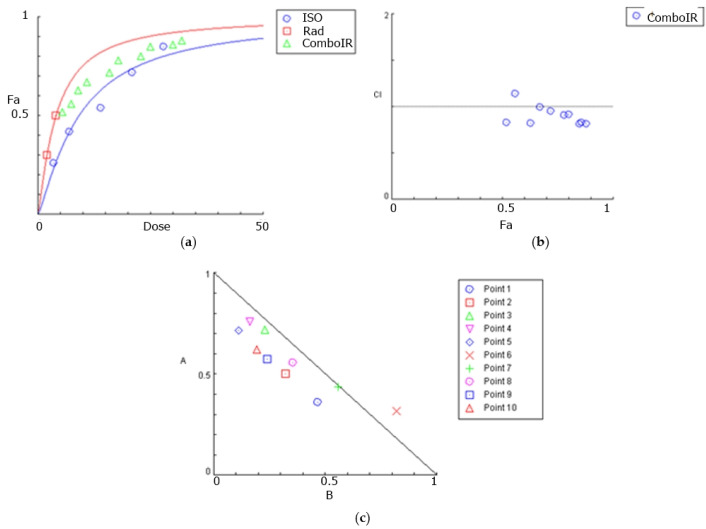
Graphical presentations obtained from the CompuSyn Report for the isocnicin and radiation combination in U87 cells: (**a**) dose–effect curve; (**b**) combination index (CI) plot for all combinations. CI = 1 defines additive effect, CI < 1 defines synergism, whereas CI > 1 is antagonism. Fa: fraction of affected cells by the drug; (**c**) isobologram: a graph indicating the equipotent combinations of various doses of two drugs and can be used to illustrate synergism, additive or antagonism. Synergism is demonstrated by the dose pair plotted as a point (symbol) below their respective line.

**Figure 5:**
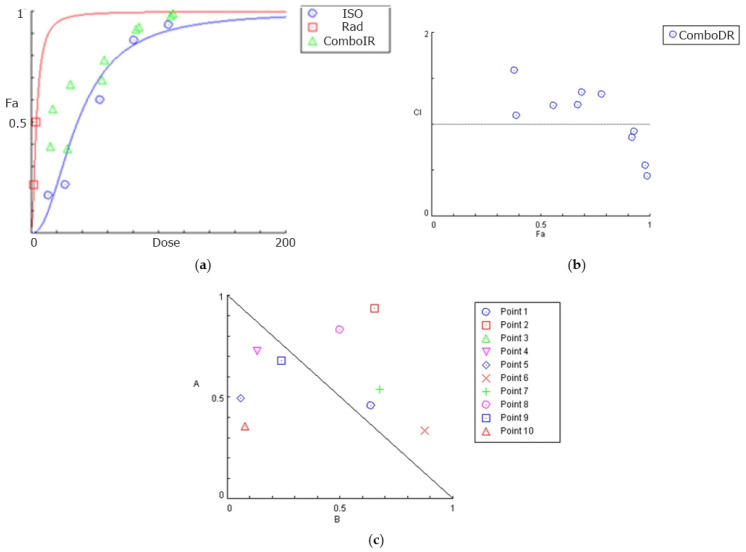
Graphical presentations obtained from the CompuSyn Report for the isocnicin and radiation combination in T98 cells: (**a**) dose–effect curve; (**b**) combination index (CI) plot for all combinations. CI = 1 defines additive effect, CI < 1 defines synergism, whereas CI > 1 is antagonism. Fa: fraction of affected cells by the drug; (**c**) isobologram: a graph indicating the equipotent combinations of various doses of two drugs and can be used to illustrate synergism, additive or antagonism. Synergism is demonstrated by the dose pair plotted as a point (symbol) below their respective line.

CompuSyn software was used to construct dose-effect curves, combination index plots, and isobolograms for the U87 (Figure 4) and T98 (Figure 5) cell lines, which graphically depict the combinatorial effects of radiation and isocnicin.

**Figure 6:**
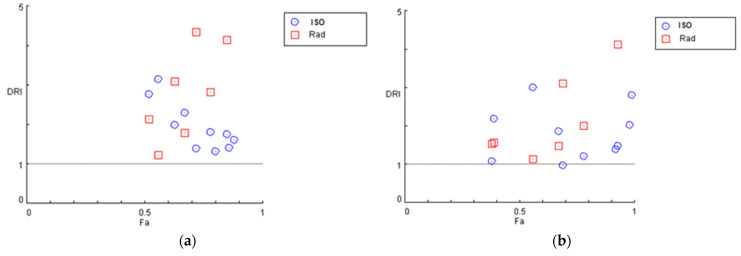
Dose reduction plots for the combination of isocnicin and radiation at different experimental points for U87 (**a**) and T98 (**b**) cells. DRI > 1 shows favorable dose reduction of both factors. Fa: fraction of affected cells by the drug.

For each drug combination, CompuSyn calculates the dose–reduction index (DRI: a measure of how many folds the dose of each drug in a synergistic combination may be reduced at a given effect level when compared with the doses of each drug alone) where DRI > 1 and <1 indicate a favourable and non-favourable dose–reduction, respectively, and DRI = 1 indicates no dose-reduction. As illustrated in Figure 6, the majority of isocnicin and radiation combinations result in a favorable dose reduction (DR > 1) in both U87 (a) and T98 (b) cell lines.

### 3.3. Isocnicin Enhanced Radiation-Induced G2/M Cell Cycle Arrest in Glioblastoma Cells

**Figure 7:**
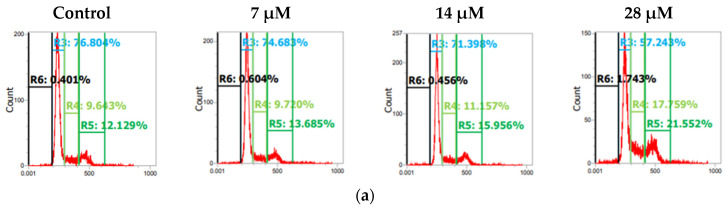

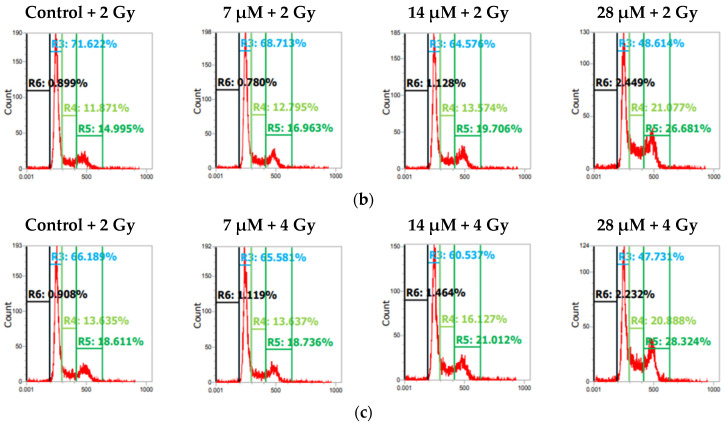
Histogram representation of the cell-cycle distribution in U87 cells after treatment with (**a**) increasing isocnicin concentrations alone (0.5 IC50 = 7 μΜ, IC50 = 14 μΜ, 2IC50 = 28 μΜ) and in combination with (**b**) 2 Gy and (**c**) 4 Gy irradiation. R6: subG0, R3: G1, R4: S, R5: G2/M.

**Figure 8:**
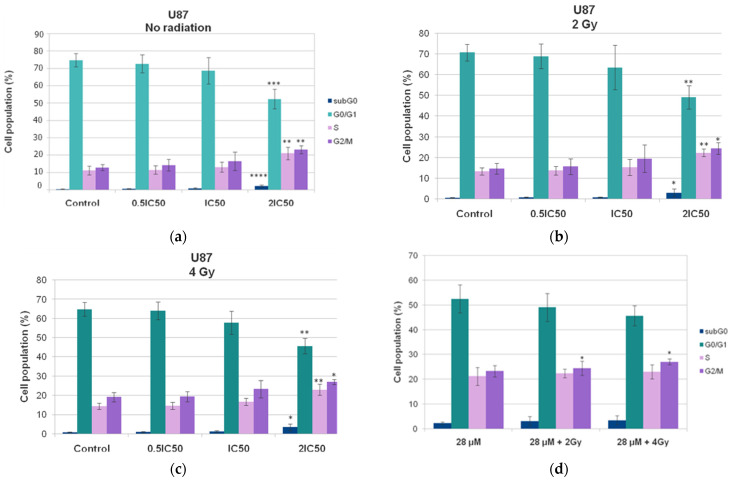
Graphical representation of cell-cycle distribution in U87 cell line after treatment with increasing isocnicin concentrations alone (**a**) and in combination with (**b**) 2 Gy and (**c**) 4 Gy radiation. (**d**) Cell cycle distribution of U87 cells after treatment with 28 μΜ isocnicin and in combination with 2 Gy or 4 Gy radiation. Data shown are the means (±S.D.) of 3 different experiments. * *p* < 0.05; ** *p* < 0.01; *** *p* < 0.001; **** *p* < 0.0001.

**Figure 9:**
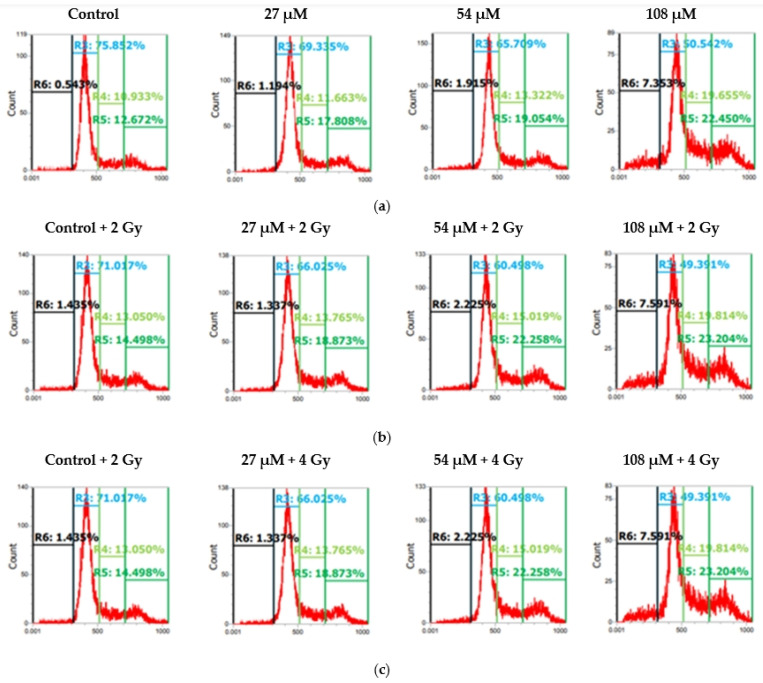
Histogram representation of the cell-cycle distribution in T98 cells after treatment with (**a**) increasing isocnicin concentrations alone (0.5 IC50 = 27 μΜ, IC50 = 54 μΜ, 2IC50 = 108 μΜ) and in combination with (**b**) 2 Gy and (**c**) 4 Gy radiation. R6: subG0, R3: G1, R4: S, R5: G2/M.

**Figure 10:**
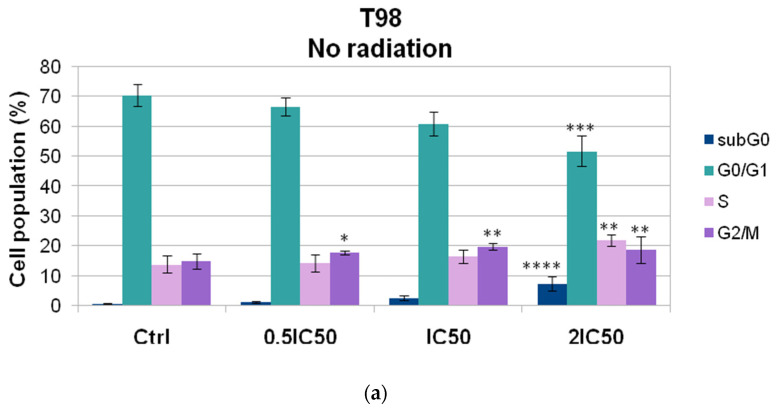

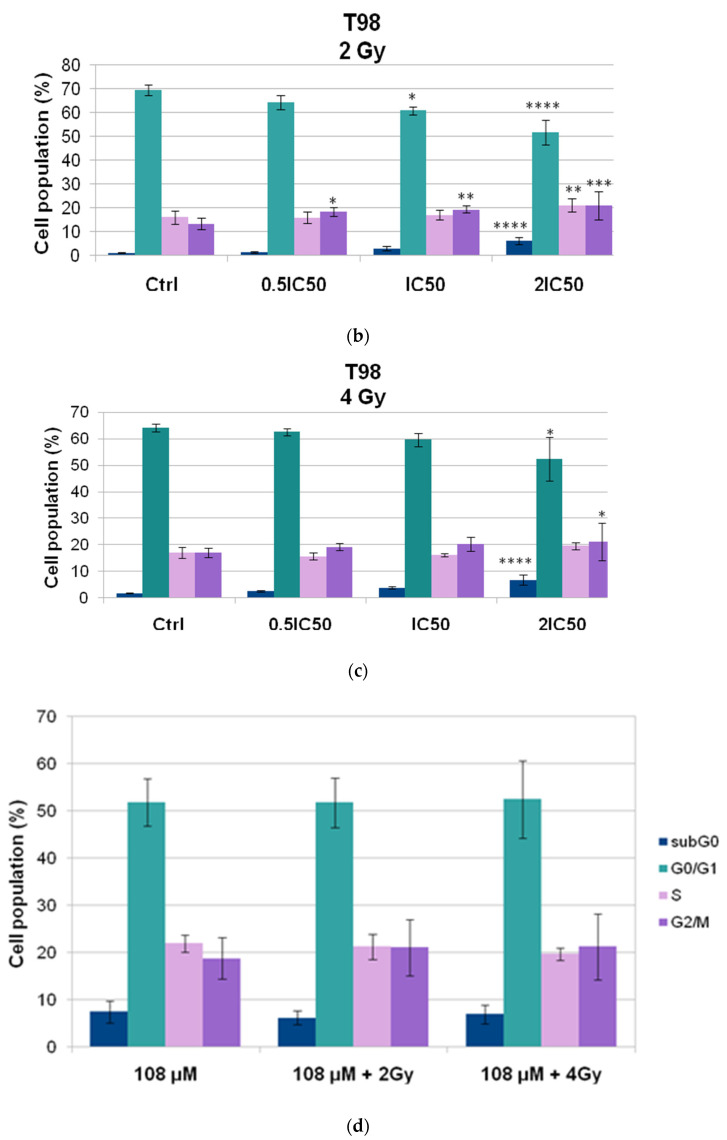
Graphical representation of cell-cycle distribution in T98 cell line after treatment with increasing isocnicin concentrations alone (**a**) and in combination with (**b**) 2 Gy and (**c**) 4 Gy radiation. (**d**) Cell cycle distribution of T98 cells after treatment with 108 μΜ isocnicin alone and in combination with 2 Gy or 4 Gy radiation. Data shown are the means (±S.D.) of 3 different experiments. * *p* < 0.05; ** *p* < 0.01; *** *p* < 0.001; **** *p* < 0.0001.

Both U87 and T98 cell lines showed substantial cytotoxicity when treated with isocnicin and radiation. Therefore, we looked at the effects of the combination treatment on several cell cycle phases. The DNA staining dye propidium iodide (PI) was used for this purpose in a flow cytometry investigation. Cell cultures were exposed to escalating isocnicin concentrations. Cells were stained with PI after 72 h, and the amount of DNA was determined. In U87 and T98 cells, isocnicin administration alone caused dose-dependent S and G2/M cell cycle arrest. Furthermore, both the U87 (Figure 7 and Figure 8) and T98 (Figure 9 and Figure 10) cell lines showed a significant increase in the percentage distribution of cells in the G2/M phase after being treated with IC50 and 2xIC50 concentrations of isocnicin and then exposed to 2 or 4 Gy of radiation. In particular, compared to treatment with isocnicin alone (23.25% ± 2.34) under the same conditions, the percentage distribution of cells in the G2/M phase (27.01% ± 1.21) was significantly improved when U87 cells were treated with 28 μM of isocnicin and then 4 Gy. In contrast to treatment with 108 μM of isocnicin alone (18.7% ± 4.44), treatment with 108 μM of isocnicin followed by 4 Gy increased the distribution of cells in the G2/M phase in the T98 cell line (21.22% ± 7.00) (Table S3). The aforementioned findings suggest that isocnicin can mitigate the harmful effects of radiation since DNA damage causes the G2/M phase to be stopped.

Based on the results so far isocnicin displayed relatively low IC50 values for both U87 and T98 cells. The compound produced S and G2/M cell cycle arrest in a dose-dependent manner. Isocnicin and irradiation showed in several cases a strong synergy relationship in both cell lines. When cells were treated with increasing concentrations of isocnicin, followed by 2 or 4 Gy of radiation, the percentage distribution of the cells in the G2/M phase was increased considerably in both cell lines

## 4. Discussion

Glioblastoma is the most common malignant primary central nervous system tumor in adults. Various chemotherapeutic agents, such as TMZ, have been used against glioblastoma. Radiotherapy and chemotherapy have a pivotal role in the treatment of glioblastoma, after surgical resection. Nevertheless, the prognosis is dismal. Still, both chemo and radioresistance were demonstrated to be a major challenge for the effective treatment of GBM [17,18,19]. Research efforts investigating novel radiosensitizers are currently underway.

Chemical agents found in plants, animals, and other natural sources are referred to as natural compounds. The molecular structures of these compounds are created by natural processes or living organisms, and they are not synthetically made. Their molecular structure and function allow them to be categorized into several groups. Alkaloids, curcuminoids, flavonoids, and coumarins are common categories of natural compounds derived from plants. They have also shown a variety of medicinal properties, including anti-inflammatory, anti-cancer, antioxidant, and analgesic activities [20]. Several natural ingredients have been found to protect glial cells from oxidative stress and neuroinflammation. They, moreover, activate apoptosis and inhibit pro-oncogene events [21,22]. Several of these agents are promising as novel anti-cancer agents [23]. Regarding gliomas, various agents displayed synergistic effects with TMZ and/or radiation [24,25]. Among them, curcumin has been extensively studied both alone and in conjunction with radiation therapy and chemotherapy drugs for the treatment of cancer [26,27,28]. Curcumin is regarded as a strong yet with fewer toxicities anti-cancer drug because, despite its ability to sensitize cancer cells to radiation, healthy cells are far less susceptible to this impact [29,30,31]. Several molecular targets of curcumin contribute to radiosensitization in different malignancies [32]. Because it is linked to the initiation, proliferation, and survival of cancer cells, the phosphatidylinositol-3-kinase (PI3K)/protein kinase B (AKT) pathway is a crucial target in cancer treatment. Through the control of several important mediators, such as growth factors, protein kinases, and cytokines, curcumin was shown to inhibit the PI3K/Akt pathway in tumor cells [33]. Other natural compounds also showed potent synergistic or antagonistic action with radiotherapy [34,35]. For example 5-Hydroxy-3′,4′,6,7-tetramethoxyflavone (TMF), a plant-derived flavone although reduced cell viability and cell migratory capacity in glioblastoma cells, there was an antagonistic effect when combined with radiotherapy. TMF-induced G0/G1 cell cycle arrest, which is believed to be a possible cause of antagonism with radiotherapy. Accumulating evidence showed that G2/M phase cell cycle arrest is one of the most important factors to enhance the sensitivity of radiotherapy [36,37].

Isocnicin is a sesquiterpene lactone isolated from the aerial parts of *Centaurea* spp. *Centaurea* species and has been used in the past to treat fever, menstrual problems, vaginal candidiasis, liver, kidney and ulcer diseases [38]. They are also known for their anti-diarrhea, stomachic, appetitive, anti-diabetic, antipyretic, and diuretic properties [39,40]. More recent studies have shown that *Centaurea* species exhibit cytotoxic and antiproliferative properties [41,42,43,44,45]. Ostad et al. showed the cytotoxic effect of *Centaurea bruguieriana* subsp. *belangeriana* against colon adenocarcinoma and breast ductal carcinoma cell lines [41]. Koukoulitsa et al. studied in vitro the cytotoxic/cytostatic activity of isocnicin against DLD1, SF268, MCF7, H460 and OVCAR3 cell lines. Among other compounds isolated from various species of the genus *Centaurea*, isocnicin was the most active [8]. In this study, we show that isocnicin reduced cell viability and induced both S and G2/M cell cycle arrest in U87 and T98 cell lines in a dose-dependent manner. When isocnicin is combined with irradiation, its anti-proliferative activity is further enhanced as the combination treatment results in increased G2/M arrest compared to isocnicin or radiation treatment alone. It is known that drugs that induce G2/M cell cycle arrest are potent radiosensitizers [46], though the mechanism of radiosensitization remains under investigation.

Glioblastoma remains the most aggressive brain tumor in adults. Current treatment includes surgical excision, chemotherapy and radiotherapy but in most cases, recurrence of the tumor is inevitable [25]. The BBB constitutes a major obstacle for glioblastoma treatment. However, isocnicin, might pass through the BBB due to its low molecular weight and lipophilic nature. The use of natural ingredients, such as isocnicin, for the treatment of glioblastoma is of great interest as these natural products might be less toxic than classic chemotherapeutic substances [47,48].

This is the first study that showed that the combination of the natural compound of *Centaurea* species, isocnicin, and radiation results in a more prominent G2/M phase cell cycle arrest compared to isocnicin or radiation alone. Until today, we knew that glioblastoma was a very aggressive and difficult-to-treat brain tumor, predominantly since the presence of the BBB is a great obstacle to chemotherapeutic drug entrance into the tumor. Thus, the need to develop novel and effective anti-cancer drugs has become a priority. Previous studies of other agents derived from plants showed low toxicity both in normal cell lines and zebrafish [49,50,51]. Thus, isocnicin deserves further evaluation in xenograft tumor models.

The current study has several limitations. First, further studies will be necessary to investigate if isocnicin can pass the BBB, such as in silico prediction. Second, the toxicity of isocnicin in normal cell lines should also be investigated. Additionally, we have not unraveled isocnicin’s mechanism of action, its synergistic effect with radiation on glioblastoma cells, and its validation in glioma xenograft models prior to clinical trials. Finally, assessing the impact of radiation 72 h after treatment may be preliminary and may require additional research employing different techniques, like a colony-forming assay, where the impact on cell viability is more clearly shown ten to fourteen days after irradiation.

In conclusion, based on the results so far, isocnicin displayed relatively low IC50 values for both U87 and T98 cells. The compound produced S and G2/M cell cycle arrest in a dose-dependent manner. Isocnicin and irradiation showed in several cases a strong synergy relationship in both cell lines. When cells were treated with increasing concentrations of isocnicin followed by 2 or 4 Gy of radiation, the percentage distribution of the cells in the G2/M phase was increased considerably in both cell lines.

## Figures and Tables

**Table 1:** Assessment of the combination effect of isocnicin and radiation in U87 cells. Combination index (CI) was determined by CompuSyn software. CI = 1 defines additive effect, CI < 1 defines synergism, whereas CI > 1 is antagonism.

Isocnicin (μΜ)	Radiation (Gy)	Effect	CI	Conclusion
3.5	2	0.52	0.82968	Synergism
7	2	0.63	0.82580	Synergism
14	2	0.72	0.95187	Synergism
21	2	0.8	0.91873	Synergism
28	2	0.86	0.82875	Synergism
3.5	4	0.56	1.13838	Antagonism
7	4	0.67	0.99624	Synergism
14	4	0.78	0.91184	Synergism
21	4	0.85	0.81458	Synergism
28	4	0.88	0.81639	Synergism

**Table 2:** Assessment of the combination effect of isocnicin and radiation in T98 cells. Combination index (CI) was determined by CompuSyn software. CI = 1 defines additive effect, CI < 1 defines synergism, whereas CI > 1 is antagonism.

Isocnicin (μΜ)	Radiation (Gy)	Effect	CI	Conclusion
13.5	2	0.39	1.09717	Antagonism
27	2	0.38	1.58854	Antagonism
54	2	0.69	1.35368	Antagonism
81	2	0.92	0.85612	Synergism
108	2	0.98	0.55446	Synergism
13.5	4	0.56	1.20989	Antagonism
27	4	0.67	1.21640	Antagonism
54	4	0.78	1.33175	Antagonism
81	4	0.93	0.92070	Synergism
108	4	0.99	0.43865	Synergism

## Data Availability

The raw data supporting the conclusions of this article will be made available by the authors on request.

## Supplementary Materials

The following supporting information can be downloaded at: https://www.mdpi.com/article/10.3390/biomedicines12122793/s1, Table S1. ^1^H-NMR of isocnicin (CDCl_3_, 500 MHz); Table S2. ^1^H-NMR of isocnicin (CD_3_OD, 500 MHz); Table S3. Cell-cycle distribution assessed by flow cytometry in U87 (a) and T98 (b) glioblastoma cell lines after treatment with isocnisin. The experiment was carried out three times and the values are means of three repetitions. * *p* < 0.05; ** *p* < 0.01; *** *p* < 0.001; **** *p* < 0.0001; Figure S1. ^1^H-NMR spectrum of 8α-O-(3′,4′-dihydroxy-2′-methylenebutanoyloxy)-dehydromelitensine (isocnicin) (CDCl_3_, 500 MHz); Figure S2. ^1^H-NMR spectrum of 8α-O-(3′,4′-dihydroxy-2′-methylenebutanoyloxy)-dehydromelitensine (isocnicin) (CD_3_OD, 500 MHz).
