# Supplementary material for: Synergistic Anti-Cancer Effects of Isocnicin and Radiotherapy in Glioblastoma: A Natural Compound’s Potential
Source: Biomedicines. 2024 Dec 9;12(12):2793. doi: 10.3390/biomedicines12122793 (PMC11673723; doi:10.3390/biomedicines12122793)
Supplement: Supplementary file 1 [file biomedicines-12-02793-s001.zip › biomedicines-3209944-supplementary.pdf]

SUPPLEMENTARY MATERIAL

Table S1. ^{1}H-NMR of isocnicin ($CDCl_3$, 500MHz).

δ (ppm)	no. H	J (Hz)	Identification
6.37	1	*br s*	H-5´a
6.13	1	*d* (J=3.2)	H-13a
6.05	1	*br s*	H-5´b
5.75	1	*dd* (J=10.8, 17.4)	H-1
5.53	1	*d* (J=2.9)	H-13b
5.40	1	*br s*	H-3a
5.27	1	*dt* (J=10.8, 4.2)	H-8
5.04	1	*d* (J=10.8)	H-2a
4.99	1	*d* (J=17.4)	H-2b
4.96	1	*br s*	H-3b
4.62	1	*dd* (J=3.4, 6.4)	H-3´
4.22	1	*t* (J=11.5)	H-6
4.07	1	*d* (J=13.7)	H-15a
3.99	1	*d* (J=13.2)	H-15b
3.83	1	*dd* (J=11.0, 3.4)	H-4´a
3.58	1	*dd* (J=11.0, 6.6)	H-4´b
2.94	1	*dddd*(J=11.0, 10.8)	H-7
2.56	1	*d* (J=11.7)	H-5
2.02	1	*dd* (J=4.2, 12.2)	H-9b
1.66	1	*dd* (J=11.5, 12.2)	H-9a
1.16	3	*s*	H-14

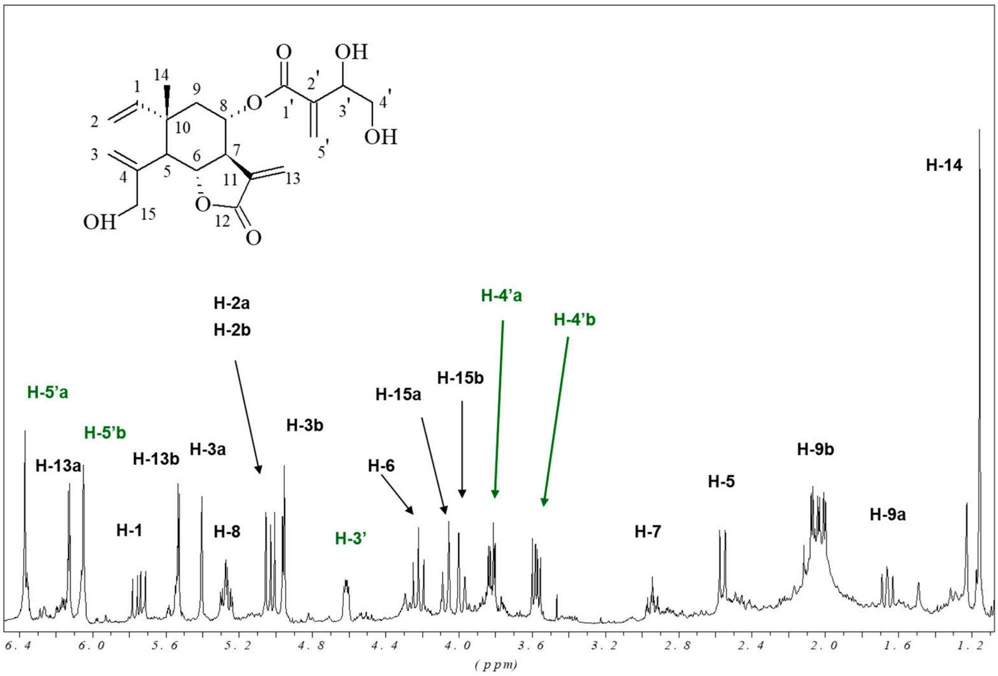

Figure S1. H-NMR spectrum of 8*α*-O-(3',4'-dihydroxy-2'-methylenebutanoyloxy)-dehydromelitensine (isocnicin) ($CDCl_3$, 500MHz).

Table S2. ^{1}H-NMR of isocnicin (CD_3OD, 500MHz).

Identification	δ_H (ppm)	No. H	J(Hz)
H-1	5.87	1	dd (J=10.9, 17.5 Hz)
H-2a	5.02-5.07	1	m
H-2b	5.02-5.07	1	m
H-3a	5.44	1	s
H-3b	5.03	1	s
H-5	2.54	1	d (J=11.8 Hz)
H-6	4.51	1	t (J=11.5 Hz)
H-7	3.10	1	tt (J=3.1, 10.9 Hz)
H-8	5.36	1	dt (J=4.1, 10.8 Hz)
H-9a	1.98	1	dd(J=4.2, 12.9 Hz)
H-9b	1.77	1	t (J= 11.5Hz)
H-13a	6.07	1	d (2.8 Hz)
H-13b	5.62	1	d (3. Hz)
H-14	1.22	3	s
H-15a	4.08	1	d (14.9 Hz)
H-15b	3.98	1	d (14.9 Hz)
H-3΄	4.61	1	t (J=5 Hz)
H-4΄a	3.72	1	dd (J=3.8, 11.2 Hz)
H-4΄b	3.53	1	dd (J= 6.5, 11.2 Hz)
H-5΄a	6.39	1	s
H-5΄b	6.08	1	s

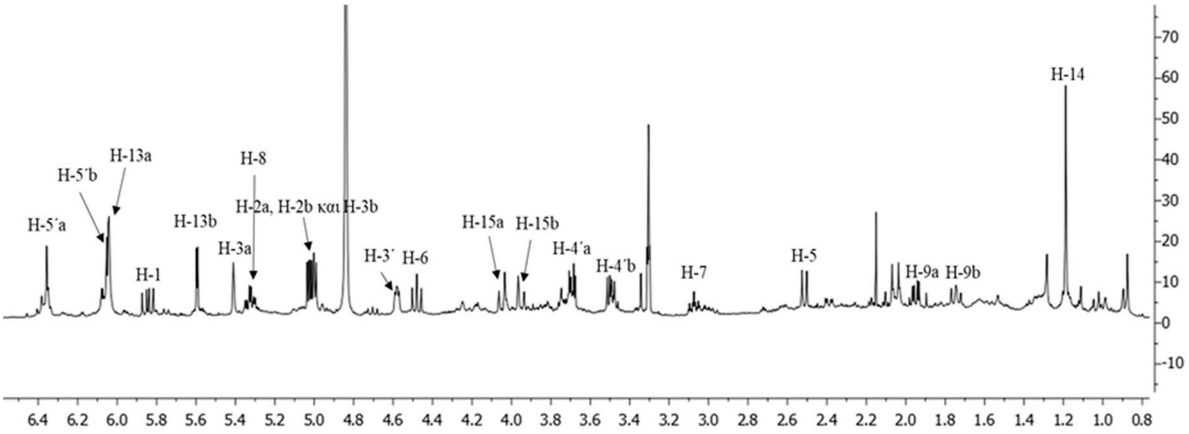

Figure S2. H-NMR spectrum of 8α-O-(3',4'-dihydroxy-2'-methylenebutanoyloxy)-dehydromelitensine (isocnicin) (CD_3OD, 500MHz).

Table S3. Cell-cycle distribution assessed by flow cytometry in U87 (a) and T98 (b) glioblastoma cell lines after treatment with isocnisin. The experiment was carried out three times and the values are means of three repetitions. * p< 0.05; ** p < 0.01; *** p<0.001; **** p<0.0001.

a

U87 cells treatment	SubG0/G1	G0/G1	S	G2/M
**Control**	0,42±0,12	74,78±3,79	11,26±2,5	13,01±1,71
**7µM Isocnicin**	0,67±0,23	72,75±5,18	11,57±2,5	14,33±3,35
**14µM Isocnicin**	0,88±0,32	68,8±7,63	13,15±2,83	16,51±5,37
**28µM Isocnicin**	2,27±0,55****	52,49±5,64***	21,13±3,58**	23,25±2,34**

**Control+2Gy**	0,63±0,17	70,63±4,07	13,38±1,7	14,71±2,64
**7µM Isocnicin+2Gy**	0,86±0,10	68,83±5,98	13,73±2,04	15,71±3,76
**14µM Isocnicin+2Gy**	0,84±0,23	63,4±10,73	15,28±3,92	19,49±6,57
**28µM Isocnicin+2Gy**	3,1±1,84*	49,05±5,63**	22,35±1,8**	24,47±2,84*
**Control+4Gy**	0,77±0,3	64,83±3,54	14,37±1,59	19,11±2,39
**7µM Isocnicin+4Gy**	0,87±0,45	64,06±4,58	14,62±1,82	19,36±2,75
**14µM Isocnicin+4Gy**	1,15±0,44	57,84±6,06	16,67±1,76	23,26±4,43
**28µM Isocnicin+4Gy**	3,4±1,82*	45,63±4,05**	22,97±2,87**	27,01±1,21*

b

T98 cells treatment	SubG0/G1	G0/G1	S	G2/M
**Control**	0,74±0,04	70,45±3,74	13,82±2,83	14,84±2,48
**27µM Isocnicin**	1,25±0,29	66,57±3,04	14,27±2,73	17,77±0,75*
**54µM Isocnicin**	2,63±0,68	60,84±3,89	16,51±2,13	19,84±1,22**
**108µM Isocnicin**	7,37±2,38****	51,79±5,04***	21,88±1,83**	18,7±4,44**
**Control+2Gy**	1,19±0,19	69,48±2,1	16,06±2,73	13,23±2,36
**27µM Isocnicin+2Gy**	1,42±0,37	64,27±2,92	16,01±2,35	18,29±1,75*
**54µM Isocnicin+2Gy**	2,82±0,93	60,83±1,62*	17,04±1,91	19,31±1,46**
**108µM Isocnicin+2Gy**	6,11±1,5****	51,73±5,27****	21,19±2,7**	20,98±5,89***
**Control+4Gy**	1,88±0,23	64,11±1,39	17,05±1,95	16,93±1,71
**27µM Isocnicin+4Gy**	2,56±0,37	62,53±1,38	15,65±1,42	19,16±1,31
**54µM Isocnicin+4Gy**	3,95±0,5	59,58±2,52	16,13±0,63	20,28±2,53
**108µM Isocnicin+4Gy**	6,87±1,97****	52,36±8,23*	19,58±1,34	21,22±7*
